# Supplementary material for: Exploring RNA-Seq Data Analysis Through Visualization Techniques and Tools: A Systematic Review of Opportunities and Limitations for Clinical Applications
Source: Bioengineering (Basel). 2025 Jan 12;12(1):56. doi: 10.3390/bioengineering12010056 (PMC11760846; doi:10.3390/bioengineering12010056)
Supplement: Supplementary file 1 [file bioengineering-12-00056-s001.zip › bioengineering-3359590-supplementary.pdf]

Supplementary Materials

Exploring RNA-seq data analysis through visualization techniques and tools: A systematic review for opportunities and limitations for clinical applications

Farhana Manzoor [1,‡] , Cyruss A. Tsurgeon [2,‡] and Vibhuti Gupta [1,*]

[1]Department of Computer Science and Data Science, School of Applied Computational Sciences, Meharry Medical College, Nashville, TN, USA; fmanzoor24@mmc.edu

[2]Department of Biomedical Data Science, School of Applied Computational Sciences, Meharry Medical College, Nashville, TN, USA; ctsurgeon07@mmc.edu

***** Correspondence: vgupta@mmc.edu

‡ These authors contributed equally to this work.

1. **Distribution of Visualization Tools by their types**

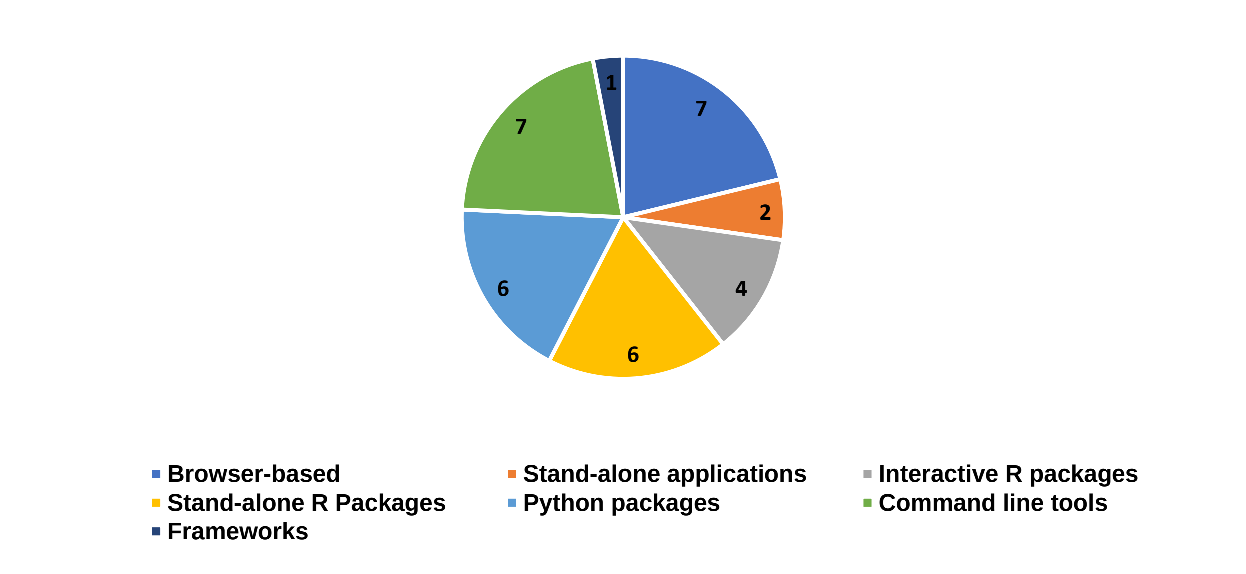

Figure S1. Distribution of Visualization Tools by their types of usage

Figure S1 shows the distribution of reviewed visualization tools by their type of usage. As shown in the above plot, most of the tools for RNA-seq visualization are browser-based or command line tools followed by R and Python packages. The detailed description of all the tools mentioned in the paper based on their types is shown below:

- **Browser-Based**: SeqCVIBE, JBrowse, scCloudMine, scViewer, CircNetVis, CircVIS, CircView.
- **Stand-Alone Applications**: Graphia Professional, BEAVR.
- **Interactive R Packages**: pcaExplorer, ideal, GeneTonic, SCUBI.
- **Stand-alone R Packages**: Searchlight, expressyouRcell, CountClust, dittoSeq, SWNE, corral, SCTK-QC.
- **Python Packages**: FIt-SNE, CP-PaCMAP, DTAE, net-SNE, autoCell, VASC.
- **Command-Line Tools**: MegaBLAST, SAMtools, CIRI-hub, CIRI-vis, INTEGRATE-Circ & INTEGRATE-Vis, VASC.

- **Algorithms/Frameworks**: Mapper

2. Distribution of visualization tools by Programming Languages and Frameworks

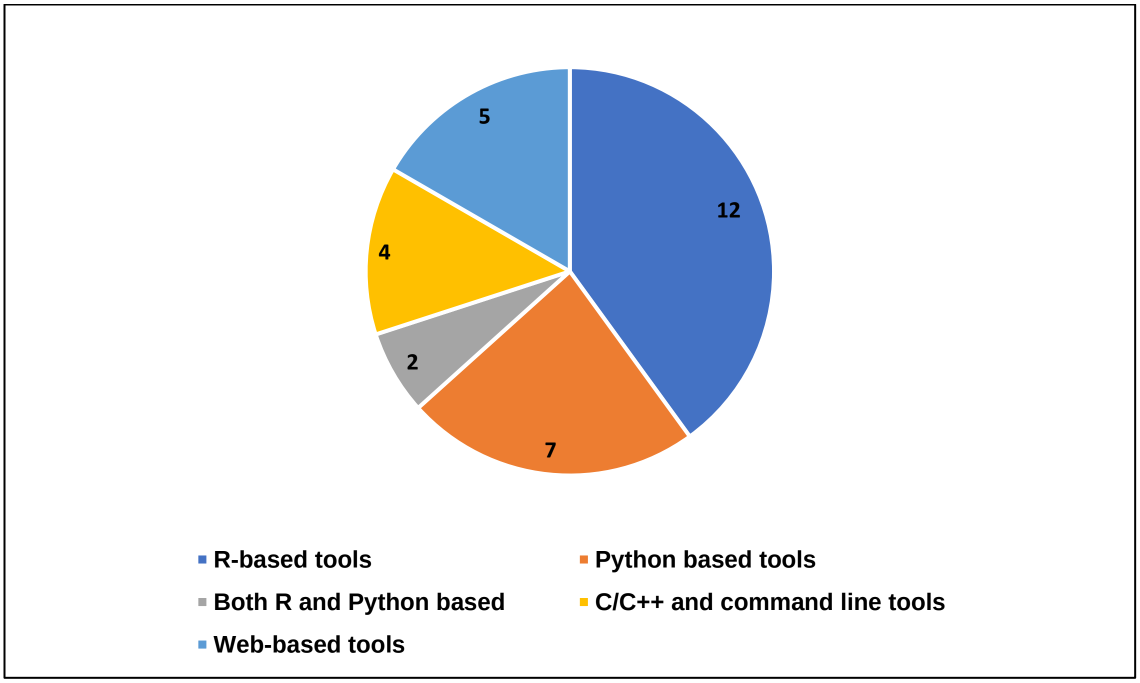

Figure S2. Distribution of visualization tools by programming languages and frameworks

Figure S2 shows the distribution of reviewed visualization tools by programming languages and frameworks. As shown in the above plot, most of the tools are R-based (12), followed by python-based tools (7) and web-based tools (5). The detailed description of all the visualization tools mentioned in the paper based on their programming languages and frameworks is shown below:

R-Based Tools:

1. Searchlight [18]
2. expressyouRcell [28]
3. CountClust [29]
4. dittoSeq [30]
5. pcaExplorer, ideal, GeneTonic [31]
6. SWNE [34]
7. corral [45]
8. SCTK-QC [50]
9. BEAVR [32]
10. Ularcirc [25]
11. ssPCA [37]
12. SCUBI [48]

Python-Based Tools:

1. VASC [33]
2. DTAE [38]
3. FIt-SNE [40]
4. CP-PaCMAP [41]
5. net-SNE [47]
6. autoCell [49]
7. Mapper: Implementations available in Python (e.g., KeplerMapper) [39]

Both R and python based:

1. WASP: Python and R [42]
2. scRNASequest: Python and R [43]

C/C++ and Command-Line Tools:

1. MegaBLAST, SAMtools [20]: Developed in C/C++
2. Graphia Professional [20]: Likely developed in C++ for performance
3. CIRI-hub [21], CIRI-vis [22]: Possibly C++ or Perl
4. INTEGRATE-Circ & INTEGRATE-Vis [23]: Likely C++ or Java

Web-Based Tools (JavaScript and Web Technologies):

1. JBrowse [19]: JavaScript-based genome browser
2. scCloudMine [35]: Web application, using JavaScript frameworks
3. scViewer [36]: Web-based tool, developed with JavaScript or Python back-end
4. CircNetVis [26], CircVIS [10]: Web applications, likely using JavaScript or Java
5. CircView [24]: Java-based

R and Python are the predominant languages used, reflecting their popularity in bioinformatics for statistical analysis and data visualization.

3. Distribution of visualization tools by the outcomes

Below is the clustering of visualization tools by their outcomes

Common Themes:

1. **Enhancing Data Visualization and Interpretation**: Many tools aim to simplify and improve the visualization of complex RNA-Seq data, making it more accessible and interpretable for researchers [18][30][32]

2. **Dimensionality Reduction and Clustering**: A significant number of tools focus on reducing the dimensionality of high-throughput data to identify patterns, clusters, and subpopulations within the data [21,33,34,37,40].

3. **Identification of Novel Biomarkers and Therapeutic Targets**: Tools are developed to detect novel RNA species like circular RNAs (circRNAs) with potential diagnostic and therapeutic implications, especially in cancer research [21-24].

4. **Handling Large and Complex Datasets**: Addressing the computational challenges associated with large RNA-Seq datasets, tools are optimized for performance and scalability [20,40,47].

5. **Integration of Functional and Pathway Analysis**: Integration of gene expression data with functional enrichment analyses to identify key pathways and gene signatures involved in diseases [31].

6. **Facilitating Single-Cell RNA-Seq Analysis**: With the rise of single-cell RNA-Seq, many tools are tailored to handle the unique challenges of scRNA-Seq data, such as sparsity and dropout events [33,36,42].

7. **User Accessibility and Interactive Exploration**: Emphasis on creating user-friendly interfaces and interactive platforms to allow researchers without extensive computational expertise to analyze and visualize data [30,35,42].

4. **Distribution of visualization methods clustered by techniques**

1. **Dimension Reduction and Clustering Techniques:**
    a. Principal Component Analysis (PCA) Plots [1,2,4,14-16,27]
    b. Multidimensional Scaling (MDS) [2,4]
    c. t-Distributed Stochastic Neighbor Embedding (t-SNE) [4,16,25,27,28]
    d. Uniform Manifold Approximation and Projection (UMAP) [4,18,25,26,28,32]
    e. Isomap, Maximum Likelihood Locally Linear Embedding (MLLE), Independent Component Analysis (ICA) [27]
    f. ssPCA Plots [20]
    g. Nonlinear Autoencoder-Based Embeddings [21]
    h. Mapper Graphs [22]
    i. Euclidean and Hyperbolic Space Visualizations [29]
    j. Neural t-SNE Embeddings [30]
2. **Differential Expression and Gene Expression Visualization:**
    a. MA Plots [1,14]
    b. Volcano Plots [1,14,15,26]
    c. Heatmaps [1,2,13-16,25]
    d. Violin Plots [19,25,26,33]
    e. Scatter Plots [13,24,33]
    f. Density Plots [33]
    g. Feature Plots [19]
3. **RNA Structure and Splicing Visualization:**
    a. Graph-Based 3D RNA-Seq Assembly Graphs [3]
    b. Circular RNA Splice Events Visualization [5]
    c. Fusion-Derived Circular RNA Visualization [6]
    d. Circular RNA Structures (Exons and Introns) [7]
    e. Sushi Genomic Visualization and Backsplice Junction Visualization [8]
4. **Functional and Network Visualization:**
    a. Interaction Network Visualization [9]

    b. Functional Annotation Visualizations [10]
5. **Genome Browser and Signal Plots:**
    a. RNA Signal Plots [2]
    b. JBrowse Visualizations [2]
6. **Quality Control and Sample Clustering:**
    a. Structure Plot for Sample Clustering [12]
    b. Grid-Based Gene Expression Visualization [31]
    c. Comprehensive Quality Control Plots [33]
7. **Specialized Visualizations:**
    a. Gene Expression Changes in Subcellular Compartments [11]
    b. 2D Embedding of Cells and Genes [17]
    c. Cluster and Values Mode, 3D UMAP Clustering [18]
